# Modeling of Cutting Parameters and Tool Geometry for Multi-Criteria Optimization of Surface Roughness and Vibration via Response Surface Methodology in Turning of AISI 5140 Steel

**DOI:** 10.3390/ma13194242

**Published:** 2020-09-23

**Authors:** Mustafa Kuntoğlu, Abdullah Aslan, Danil Yurievich Pimenov, Khaled Giasin, Tadeusz Mikolajczyk, Shubham Sharma

**Affiliations:** 1 Mechanical Engineering Department, Technology Faculty, Selcuk University, Selçuklu, Konya 42130, Turkey; 2 Mechanical Engineering Department, Engineering and Architecture Faculty, Selcuk University, Akşehir, Konya 42550, Turkey; aaslan@selcuk.edu.tr; 3 Department of Automated Mechanical Engineering, South Ural State University, Lenin Prosp. 76, 454080 Chelyabinsk, Russia; 4 School of Mechanical and Design Engineering, University of Portsmouth, Portsmouth PO1 3DJ, UK; Khaled.Giasin@port.ac.uk; 5 Department of Production Engineering, UTP University of Science and Technology, Al. Prof. S. Kaliskiego 7, 85-796 Bydgoszcz, Poland; tami@utp.edu.pl; 6 Department of Mechanical Eng., IKG Punjab Technical University, Jalandhar-Kapurthala Road, Kapurthala, Punjab 144603, India; shubham543sharma@gmail.com

**Keywords:** vibration, surface roughness, turning, response surface methodology, analysis of variance

## Abstract

AISI 5140 is a steel alloy used for manufacturing parts of medium speed and medium load such as gears and shafts mainly used in automotive applications. Parts made from AISI 5140 steel require machining processes such as turning and milling to achieve the final part shape. Limited research has been reported on the machining vibration and surface roughness during turning of AISI 5140 in the open literature. Therefore, the main aim of this paper is to conduct a systematic study to determine the optimum cutting conditions, analysis of vibration and surface roughness under different cutting speeds, feed rates and cutting edge angles using response surface methodology (RSM). Prediction models were developed and optimum turning parameters were obtained for averaged surface roughness (*R_a_*) and three components of vibration (axial, radial and tangential) using RSM. The results demonstrated that the feed rate was the most affecting parameter in increasing the surface roughness (69.4%) and axial vibration (65.8%) while cutting edge angle and cutting speed were dominant on radial vibration (75.5%) and tangential vibration (64.7%), respectively. In order to obtain minimum vibration for all components and surface roughness, the optimum parameters were determined as *V_c_* = 190 m/min, *f* = 0.06 mm/rev, *κ* = 60° with high reliability (composite desirability = 90.5%). A good agreement between predicted and measured values was obtained with the developed model to predict surface roughness and vibration during turning of AISI 5140 within a 10% error range.

## 1. Introduction

AISI 5140 is a medium carbon steel which is widely used in the automotive industry. The alloy is used in other applications such as marine engineering, furnaces, gas turbines, chemical processing plants, and pressure vessels. Despite its wide range of applications, the high content of chromium in AISI 5140 generates high structured carbides making it difficult to machine. Alsaran et al. [1,2,3] investigated the mechanical properties, structural characterization and tribological properties of nitride AISI 5140 low-alloyed steel. Grzesik [4] explored the wear mechanisms of ceramic inserts during hard turning of AISI 5140 steel. Other authors investigated the tool wear and chip morphology during turning of AISI 5140 [5], tool wear during boring [6], surface roughness and cutting forces [7] and surface roughness and tool wear [8]. To ascertain the machining performance of AISI 5140, Huang et al. [9] studied the influence of lubrication on surface quality in grinding. Grzesik and Wanat [10] examined the surface roughness during hard turning. On machining optimization of AISI 5140, Kahraman [11] optimized the cutting parameters for surface roughness using a Taguchi method. Kuntoğlu et al. [12] applied an optimization and analysis approach using a tool condition monitoring system in turning of AISI 5140. Lastly, optimization and analysis of process parameters for flank wear, cutting forces and vibration have been performed and it was found that these quality indicators were correlated with each other and statistically reliable [13]. There are limited studies in the open literature and only a handful of them investigated the machinability and optimization of AISI 5140 steel alloy. The paper aims to fill this gap in the literature.

Surface roughness reflects the surface quality of a product in generally accepted terms [14]. The most preferred characteristic to determine the quality of surface roughness is the mean roughness *R_a_* [15]. *R_a_* value is determined according to workpiece specifications which can be detailed by the producer or the consumer [16]. The desired surface quality of a machined part can be achieved by finding the optimum cutting conditions [17]. A good surface finish provides better mechanical properties for a machine element related to the useful remaining tool life [18,19,20,21,22]. Surface roughness is a feature of the outer form of the machined material and which can be controlled to obtain certain functional properties such as friction, thermal conductivity and oil retention [23]. The surface roughness value is commonly required to fall within a certain range based on the final application of the machined part [24].

In the turning process, the most affecting parameters on surface roughness are the feed and the tool nose radius [25]. However, the complex structure of machining processes and the dynamic interaction of the machine and cutting tool parameters increase the effect level of other parameters depending on the structure of the process [26]. In a study by Abbas et al. [27] it was observed that the effect of cutting speed on surface roughness was greater than that of the feed rate

In machining, vibration occurs due to a lack of rigidity in the machine tools and cutting tool clamping or due to changing cutting conditions during the cutting process [28]. Vibration is an undesirable phenomenon that negatively affects the cutting process [29]. In general terms, vibration can be described as an oscillation around an equilibrium point which occurs in the form of disruption of the contact area between the tool geometry originally determined for cutting and the workpiece [30]. During the relative movement of the cutting tool and the workpiece, undesirable results such as the loss of the theoretically determined tool geometry occur and thus the surface form of the workpiece is deformed [31].

Modeling and optimization are two significant tools used to perform robust analysis and cost-effective approaches in high precision manufacturing [32,33]. The relationship between process variables can be obtained via mathematical equations and the correlation between them can be determined with modeling. Response surface methodology (RSM) uses multiple regression models to carry out statistical analysis of a system. Sarıkaya and Güllü [34] investigated the effect of cutting parameters and cooling conditions based on Taguchi, RSM and Analysis of Variance ANOVA during turning of AISI 1050 steel. Chauhan [35] compared the success of modeling of artificial neural networks (ANN) and RSM in turning of the hybrid composite material. Yadav et al. [36] proposed a hybrid approach of Taguchi–RSM for improving the surface roughness in turning. Because of the availability and advantage of these two approaches in modeling and optimization in complex processes, it was preferred, and an important improvement was obtained. RSM was also implemented in past studies during the turning of metal matrix composites and AISI 1045 steel [37,38]. Thomas et al. studied the impact of feed rate, tool radius and vibration on surface roughness and found that only the feed rate and tool radius had an impact on surface roughness [39]. Sajjady et al. [40] analyzed the effect of the cutting speed, feed rate and vibration process on surface roughness and found that feed rate was the dominant factor (75.38%) followed by vibration process (5.7%). Makadia et al. [14] studied the impact of tool nose radius and cutting parameters using RSM during turning of AISI 410 steel; the study showed that the feed rate was the most influential factor on surface roughness. Bouacha et al. [41] investigated the optimum conditions for surface roughness and cutting forces using RSM. In turning of Hadfield steel, RSM was employed for modeling and analysis of machining parameters for surface roughness [42]. Parida and Maity [43] determined the optimal cutting conditions via RSM in turning of Monel 500 with 86.7% composite desirability. Abbas et al. [44] employed RSM optimization in turning of AISI 1045 steel for investigation of surface roughness. On the investigation of surface roughness of AISI 5140 steel, the effect of cutting parameters namely feed rate and depth of cut [8,10,11] and cutting speed [7,8,11] were incorporated into the experimental plan. However, the influence of cutting edge angle was not reported in the past studies which motivate the need to study its impact on surface roughness linearly and interactively in the current study. From the reported studies, based on [14,34,35,41] feed rate was a major factor in surface roughness; however, the papers [35,42,43,44] showed that tool tip and cutting speed were the major factors affecting surface roughness. Contrary to the known theoretical assumption that explains the effect of feed rate on surface roughness, due to the interactions between input parameters and unexpected developments such the existence of different types of tool wear, the dominant parameters show an alteration.

Prasad and Babu [28] investigated the effect of cutting parameters on vibration with ANOVA and they reported that feed rate had a great influence on chatter vibration. Ozbek et al. [45] reported that all axes of vibration amplitude increase with the increase of cutting speed during turning of AISI D2 steel. The contradiction between these two papers [28,45] arises from the complexity of vibration and the complex triggering mechanism behind it. As a result, different cutting parameters can be effective which change according to the determined experimental plan and ranges of parameters. Plaza et al. [46] stated that among three vibration components, axial acceleration was the reliable source among others for monitoring of surface roughness. Wang et al. [30] presented an approach using vibration signals to predict surface roughness separating the frequency as high and low. High frequency vibration on the tool tip dominantly affected surface roughness. He et al. [31] demonstrated that increasing the amplitude of tool tip vibration enhances surface roughness. According to Abouelatta and Madl, during turning [47], surface roughness can be estimated using cutting parameters and radial and axial components of vibration. Risbood et al. [48] studied the effect of cutting parameters on the developed cutting forces and vibrations and used that to predict surface roughness via artificial neural network systems. Misaka et al. [49] developed a new method for predicting surface roughness using cutting parameters and vibration signals via RSM. Upadhyay et al. [50] carried out a study to predict surface roughness by varying the feed rate, depth of cut, tangential and radial vibration components using ANN. Hesseina et al. [51] presented a study based on RSM to predict surface roughness utilizing radial and tangential vibration as well as cutting parameters. The outline of the past studies investigating the effects of cutting parameters on surface roughness and vibration components are listed in Table 1. It should be noted that none of the above studies investigated the effect cutting parameters on vibration, optimization and correlation between vibration and surface roughness for AISI 5140.

Based on the previous literature on turning AISI 5140 steel alloys, the current paper’s aim is to study the effect of cutting parameters (cutting speed, feed rate and cutting edge angle) on the machined surface roughness and vibration in three directions when turning AISI 5140 steel. In addition, multi-criteria optimization was employed to establish a relationship between surface roughness and vibration components. Quadratic regression models and multiple optimization of surface roughness and the three components of vibration were achieved via RSM. Lastly, the three components of vibration were compared according to experiments to understand the relationship between vibration and surface roughness. The study is different from the past studies since it provides simultaneous optimization and relationship between surface roughness and vibration in additional to analyzing them individually.

## 2. Materials and Methods

### 2.1. Workpiece Material and Cutting Tools

In this study, an AISI 5140 steel rod of Ø75 mm and 500 mm in length was chosen as workpiece material which is a common size standard in industrial applications and hard-to-wear structures. The chemical composition of the material is presented in Table 2. Experiments were carried out on the lathe (De Lorenzo S547-8899, Milano, Italy) under dry cutting conditions. The depth of cut was kept constant at 2 mm. Coated carbide cutting tools which are suitable for machining metallic alloys were used in the study. The tools are commonly used in more than 80% of the studies since they were reported to provide better surface roughness [52].

A new workpiece material was utilized in every experiment and four pass chips were removed with an insert and each experiment was repeated three times. According to the manufacturer’s handbook and machine tool operation range, cutting parameters were selected with three cutting speeds, three feed rates and three cutting edge angles as shown in Table 3.

### 2.2. Experimental Study

**Figure 1:**
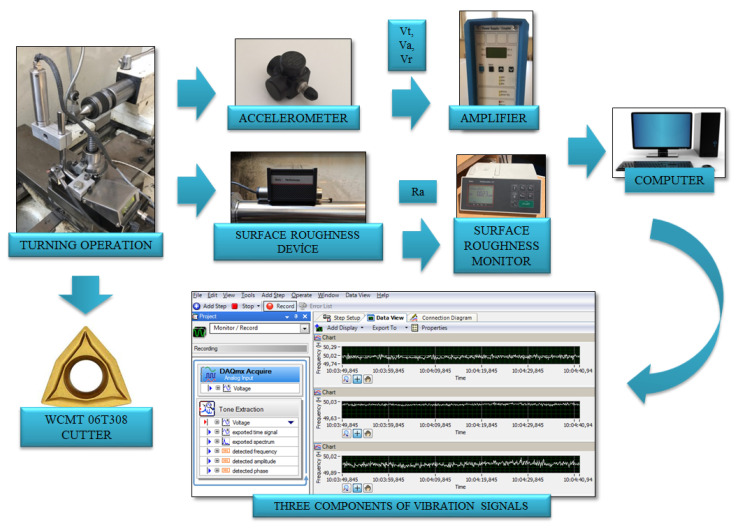
Experimental setup.

The experimental setup includes the machine tool, measuring devices, sensors, data acquisition units and a computer. The experimental setup is shown in Figure 1. For measuring the vibration, an accelerometer which can sense the three components of acceleration was utilized. The accelerometer (Kistler 8692C50, Winterthur, Switzerland) can be mounted on the machine parts using the magnetic part at its base. In addition, it can be permanently fixed on the free surfaces with its special adhesive. The accelerometer is attached to an amplifier (Kistler 5134B) for accommodation and compensation of data before data transmitting. Vibration data were transmitted to the computer via the data acquisition card (National Instruments USB-6003, Austin, TX, USA), the processing and recording of the signals were performed via Signal Express software.

**Figure 2:**
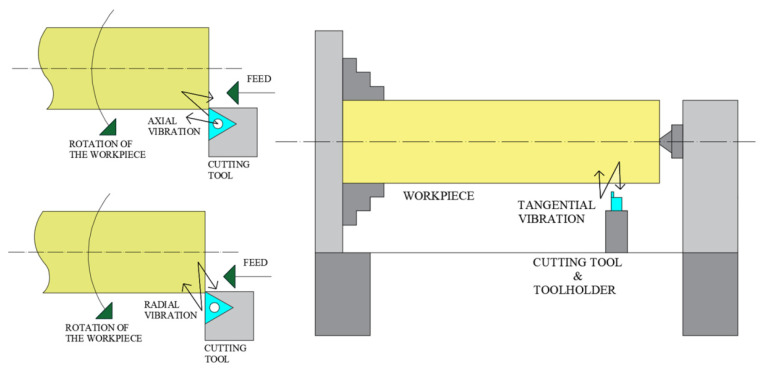
Vibration components occurring on the machine tool.

Generally, three vibration components are measured from machine tool which can be defined as tangential vibration (*V_t_*), axial vibration (*V_a_*) and radial vibration (*V_r_*) according to the direction of vibration with respect to the cutting tool. Three components of vibration are demonstrated in Figure 2.

The surface roughness measurement was carried out with perthometer (Mahr M1, Göttingen, Germany). For each experiment, four specimens were machined using one tool tip under the same cutting conditions to investigate surface roughness. At the end of two passes, the operation was stopped, surface roughness was measured three times round the workpiece at equal distances for each experiment. Each measurement was repeated three times to confirm the repeatability. Therefore, all the data reported hereafter are the average value of the three measurements. The arithmetic mean value of the profile, *R_a_*, was selected to reflect surface roughness, which is a widely used roughness metric in production.

### 2.3. Response Surface Methodology

RSM allows the optimization of multiple parameters simultaneously and the effect of linear, square and interaction of parameters can be determined [25]. The statistical analysis was carried out using the ANOVA statistical technique which is a commonly used method to evaluate whether certain machining parameters have an impact on the analyzed outputs [53]. Using multiple regressions, the prediction of a quality characteristic is possible with high reliability [43]. There is a necessity to design the RSM model which consists of at least three factors for each control parameter [54]. This approach originated from the purpose of estimating the control parameter values which are not added to experimental design. The RSM model refers to the functional equation to correlate the control parameter and quality characteristic. Generally, a treatment approach is operated to find out the optimal solution which is described below:

*Z* = *f* ((*V_c_*), (*f*), (*κ*)) + *Error* (1)

Quadratic regression is developed where *Z* represents the response parameter namely surface roughness, *V*, *f* and *κ* represent the cutting speed, feed rate and cutting edge angle, respectively. This equation is transformed into a quadratic multiple regression model:

(2) $$Z\;=\;C_{0}\;+\;\sum_{i=1}^{3}C_{i}X_{i}\;+\;\sum_{i=1}^{3}C_{ii}X_{i}^{2}\;+\;\sum_{i<j}^{3}C_{ij}X_{i}X_{j}$$

where *C*_0_ is constant, *C_i_*, *C_ii_* and *C_ij_* are linear, square and interaction coefficients, respectively. *X* represents the evaluated control factors namely cutting speed (*V*), feed rate (*f*) and cutting edge angle (*κ*). The equation can be stated as:

*Z* = *C*_0_ + *C*_1_*V_c_* + *C*_2_*f* + *C*_3_*κ* + *C*_11_*V_c_*^2^ + *C*_22_*f*^2^ + *C*_33_*κ*^2^ + *C*_12_*V_c_f* + *C*_23_*f κ* + *C*_13_*κV_c_* (3)

### 2.4. Analysis of Variance

ANOVA implies the significance of design parameters on the investigated response parameter over diverse statistical value [17]. The importance of design or control parameters can be determined and confirmed via these statistical parameters [55]. *p*-value means the probability of significance for each control parameter and the highest value signifies the effectiveness of that parameter. The sum of the squares of the quality characteristic is calculated below with the mean value and the difference of the result of each experiment [36]. While each design parameter has a certain effect on this total, the remaining result from the sum of these effects gives the error. By dividing the sum of the squares belonging to the parameters to the sum of the sum of squares, the amount produced as a percentage of that parameter is calculated (Percent Contribution (PC) %).

### 2.5. Quadratic Regression Models

Quadratic regression aims to find the best data which are proper for the equation of a parabola. In other words, the relationship between two different variables can be stated with a parabola on the graph. In this way, a correlation between two different data can be produced. This permits the making of predictions about the handled data. The prediction power of a quadratic regression model is signified with the determination coefficient (*R*^2^) [35]. The *R*^2^ value changes within the range of 0–100% and demonstrates high accuracy of prediction as it increases [44]. Because of the high costs and challenges in performing of machining experiments, the generated model is valid under the determined cutting conditions of turning of AISI 5140 steel.

## 3. Results and Discussion

**Figure 3:**
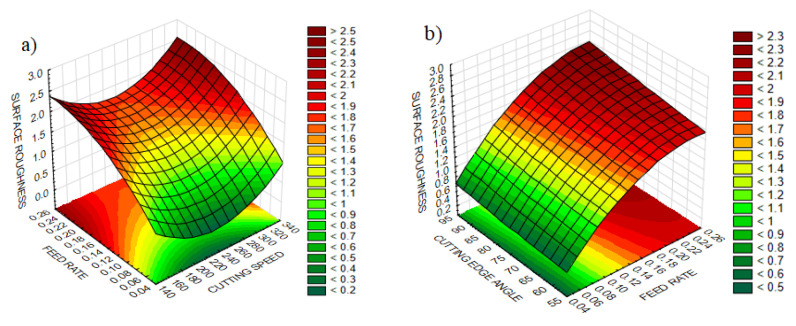
The effect of parameters on surface roughness (**a**) Combination of feed rate and cutting speed (**b**) Combination of cutting edge angle and feed rate.

**Figure 4:**
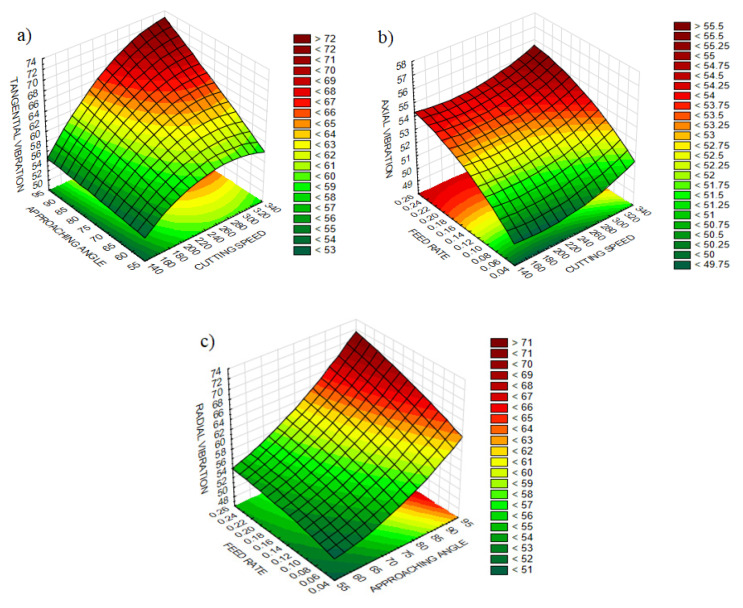
The influential parameters on 3 components of vibration (**a**) The effect of cutting edge angle and cutting speed on tangential vibration, (**b**) The effect of feed rate and cutting speed on axial vibration, (**c**) The effect of cutting edge angle and feed rate on radial vibration.

**Figure 5:**
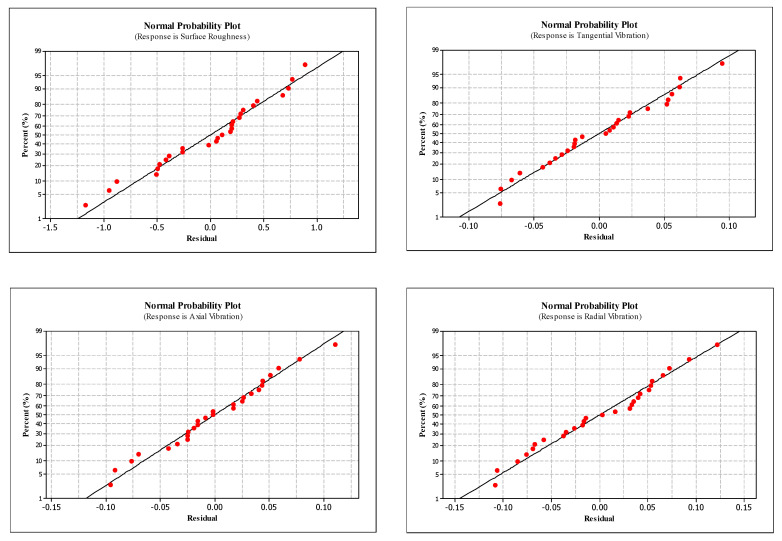
Normal probability plots for surface roughness and vibration.

**Figure 6:**
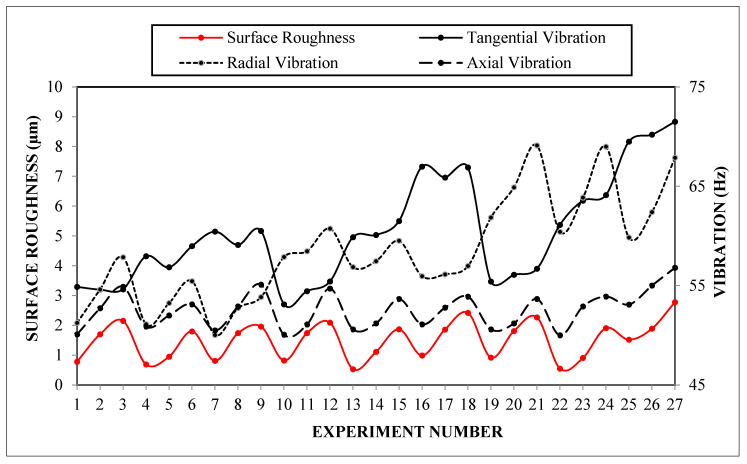
Interaction between surface roughness and vibration components.

The experimental design contains 27 experiments which represent a full factorial design. The main advantage of this approach is observing the effect of each design parameter completely on the quality indicator. Table 4 comprises the design parameters such as cutting speed, feed rate and cutting edge angle and related quality indicators namely surface roughness, and three components of vibration for each experiment. Table 5 and Table 6 represent the ANOVA results for the surface roughness and vibration components, respectively. Lastly, it is shown that the design, optimization and confirmation results of RSM are confirmed. The affecting parameters on surface roughness and vibration components are indicated in Figure 3 and Figure 4, normal probability plots for these results are shown in Figure 5. Figure 6 shows the interaction between surface roughness and vibration components for every experiment.

### 3.1. The Effect of Cutting Parameters and Tool Geometry on Surface Roughness

In order to determine the most affecting parameter, ANOVA was carried out with an RSM approach. The linear and interactive effects of the cutting parameters and cutting edge angle are tabulated in Table 5. As is expected from the popular formula [56] which is given in (4), the change in surface roughness is proportional to the square of the feed rate. The analysis result of the surface was found to be reliable as a 95% confidence interval appears. Furthermore, the analysis demonstrates clearly that the parameters of cutting speed as linear (5%) and as a square (13.2%) are the other affecting parameters on surface roughness. It was reported that cutting speed had an impact on surface roughness, altering the mechanical properties of workpiece and chip formation [57].

(4) $$R_{a}=f^{2}/32\;\cdot \;r$$

Figure 3a,b shows the impact of cutting speed, feed and cutting edge angle on surface roughness. Considering the feed rate as the most influential factor, the change with two other parameters on surface roughness was investigated. It is observed that the increase in feed rate reduces the surface roughness. It is expected that the broader helicoidal groove which arises from a higher feed rate will eventually raise the surface roughness [15]. On the other hand, increasing the cutting edge angle slightly increases the surface roughness, which can be ignored. A higher cutting edge angle leads to the sudden entrance of cutting tool into the workpiece which causes elevated cutting forces; the material becomes hard to cut and eventually increases in surface roughness [33]. However, the surface roughness curve shows a decreasing trend first and increases after that with enhancing cutting speed. Because the hardness of the workpiece reduces with high cutting speed then surface roughness decreases, generating the desired shaped chips [58] until a determined cutting speed value. After that point, accelerating tool wear arising from high cutting speed escalates surface roughness again [15]. The highest value of surface roughness is observed at the highest values of feed rate, cutting speed and cutting edge angle used in the study.

### 3.2. The Effect of Cutting Parameters and Tool Geometry on Vibration

According to the results, the percentage contributions of cutting speed, cutting edge angle and feed rate were found to be 64.7%, 75.5% and 65.8% on tangential, radial and axial vibrations, respectively. The next contributing parameter on vibration components was the cutting edge angle (19.1%), followed by the feed rate (15.7%) and cutting speed (11.7%) which is demonstrated in Table 6. It is assumed that vibration components on tangential and axial directions originated from the relative motion between tool and workpiece which can be attributed to cutting force components. Since these two vibration components are generated based on the direction of cutting speed and feed rate, the dominant effect of these parameters is understandable. Cutting edge angle specifies the distribution of cutting forces on different directions which have an impact on shear zones, contact conditions between tool and workpiece, and eventually vibration [12]. It can be said that the interaction between parameters has no significant effect on vibration components. *p* values showed that cutting speed (0.000 < 0.05 for *V_t_* and *V_r_*, 0.001 < 0.05 for *V_a_*), feed rate (0.000 < 0.05 for *V_r_* and *V_a_*, 0.014 < 0.05 for *V_t_*) and cutting edge angle (0.000 < 0.05 for *V_t_* and *V_r_*, 0.032 < 0.05 for *V_a_*) have significant effects on vibration components, illuminating the complexity and dynamic structure of vibration in turning.

Based on ANOVA, the parameters effective on vibration components are presented in Figure 4. It can be seen in Figure 4a that tangential vibration shows a rising trend with enhancing cutting speed and the rate of increase strengthens with increasing cutting edge angle. Since the cutting speed and tangential vibration occur in the same direction, increase in cutting speed improves acceleration due to the accumulation of chrome and related hard carbides. Additionally, chrome ingredients cause adhesive wear on the rake face of the cutting tool [5], which eventuates the rise of tangential vibration. It can be also that higher feed rate values accelerate axial vibration for all cutting speeds, which is demonstrated in Figure 4b. Similarly to the effect of cutting speed on tangential vibration, a high content of chrome disorders the stability of the cutting tool and tends towards oscillation. On the graph of radial vibration, it can be seen in Figure 4c that advancing cutting edge angle significantly improves radial vibration and this impact increases with higher feed rate values. Cutting edge angle settles the separation of cutting forces additional to feed rate, depth of cut and specific cutting force. In this context, an increase in cutting edge angle enhances the axial cutting force. However, variation in the feed rate dominates this direction and can alter the effect of cutting edge angle on studied outputs. As a result, increasing the cutting edge angle demonstrates its effect on the radial direction and triggers the vibration.

### 3.3. Quadratic Regression Models for Surface Roughness and Vibration

Predicted regression equations for surface roughness and three components of vibration were calculated using design parameters in Equations (5)–(8). The Equations provide valuable information about the turning process which defines the effectiveness of parameters individually and interactively. RSM was utilized to generate the quadratic mathematical models and the maximum errors of 5%, 2%, 3% and 8% of surface roughness, tangential vibration, radial vibration and axial vibration. The estimated values were found to be statistically close to the experimentally measured values.

*R_a_* = 5.31201 − 0.0422831 · *V_c_* + 15.4801 · *f* − 0.0285486 · *κ* + 0.0000757170 · *V_c_*^2^ − 34.2593 *· f*^2^ + 0.0000691358 · *κ*^2^ − 0.000837317 · *V_c_* · *f* + 0.0000946589 · *V_c_* · *κ* + 0.0289683 · *f* · *κ* (5)

*V_t_* = 39.7323 + 0.122155 *· V_c_* − 20.1914 · *f* − 0.131809 · *κ* − 0.000378965 · *V_c_*^2^ + 32.6132 · *f*^2^ − 0.000572840 · *κ*^2^ − 0.0150549 · *V_c_* · *f* + 0.00161231 · *V_c_* · *κ* + 0.274471 · *f* · *κ* (6)

*V_r_* = 64.8713 − 0.0449595 · *V_c_* + 8.92157 · *f* − 0.448740 · *κ* + 0.0000673219 · *V_c_*^2^ − 55.0412 · *f*^2^ + 0.00478272 · *κ*^2^ − 0.0388988 · *V_c_* · *f* + 0.0000696053 · *V_c_* · *κ* + 0.590212 · *f* · *κ* (7)

*V_a_* = 72.3570 − 0.0641861 · *V_c_* + 45.0033 · *f* − 0.506884 · *κ* + 0.0000707312 · *V_c_*^2^ − 60.7510 · *f*^2^ + 0.00276296 · *κ*^2^ − 0.0106063 · *V_c_* · *f* + 0.000523016 · *V_c_* · *κ* − 0.0550265 · *f* · *κ* (8)

Normal probability plots of residual values and prediction success for surface roughness and vibration components are presented in Figure 5. The adequacy of the model was verified when plotting the normal probability plots [41]. The deflection from a straight line refers to separation from normality. Half of the data points occur on the left and the other half on the right side of the straight line. The general view of the graph is specified with a line accepted to be approximately straight and separating the screen into two parts based on the data points located on it. It is shown by the graph that the data points pursue the straight line which demonstrates the proposed model is sufficient to show the suitability. All of the four graphs referring to surface roughness and three-dimensional vibrations demonstrate a similar structure. According to the graphs, the normal probability plot shows a reasonably linear pattern.

Considering the developed regression models and their adequacy, the experimentally obtained data such as cutting forces, vibration or acoustic emission in machining operations can be useful for observing the condition of the cutting tool and cutting process. Figure 6 contains the data of surface roughness and vibration components for each experiment. By taking into consideration the sensor signals of acceleration and surface roughness measurement, data points were marked in order to observe the similarity. There have been several attempts to predict surface roughness via vibration signatures. Axial and radial vibration was used [47] and radial vibration was also utilized to estimate the surface roughness [48]. In another work [46], axial vibration provided the best information for estimating the surface roughness. It was difficult in this study to compare the vibration components by reflecting upon the capability of surface roughness. According to the curves in Figure 6, axial vibration demonstrates close behavior to surface roughness variation which can be attributed to the effect of feed rate. The dominant effect of feed rate on both axial vibration and surface roughness provided the information to find the similarity. For a whole experimental plan, surface roughness and axial vibration curves pursue a similar decreasing or increasing path. It is also remarkable that the minimum frequency ranged vibration component—the axial—indicates a proximate characteristic to surface roughness. The radial vibration component on the other hand, has promising behavior for successfully monitoring the surface roughness during the first nine experiments, namely turning at low cutting edge angle values. However, tangential vibration has no beneficial notion regarding the surface roughness during turning of AISI 5140 steel.

### 3.4. Response Surface Methodology Based Optimization

The general intention in an experimental study is to find out the optimal conditions for obtaining the desired results in terms of the quality criteria [35]. RSM was utilized for the investigation of the relationship between inputs and outputs to achieve the optimum results [12]. In this paper, the attempt was to determine the optimum cutting speed, feed rate and cutting edge angle for minimum surface roughness and vibration components. To achieve this, RSM was used since it is commonly employed in machining studies and can give accurate results for single or multiple analyses [14]. Table 7 indicates the RSM parameter design and related predicted responses along with their desirability. To achieve minimum surface roughness and vibration, targets are selected as lower values from the experimental design table. As a result, a multi-criteria optimization approach was selected to obtain the optimum parameters. The obtained desirability concerning surface roughness (0.99), tangential vibration (0.77), radial vibration (0.91), axial vibration (0.95) and composite (0.9) show the accuracy of the model, however, needs to be verified.

**Figure 7:**
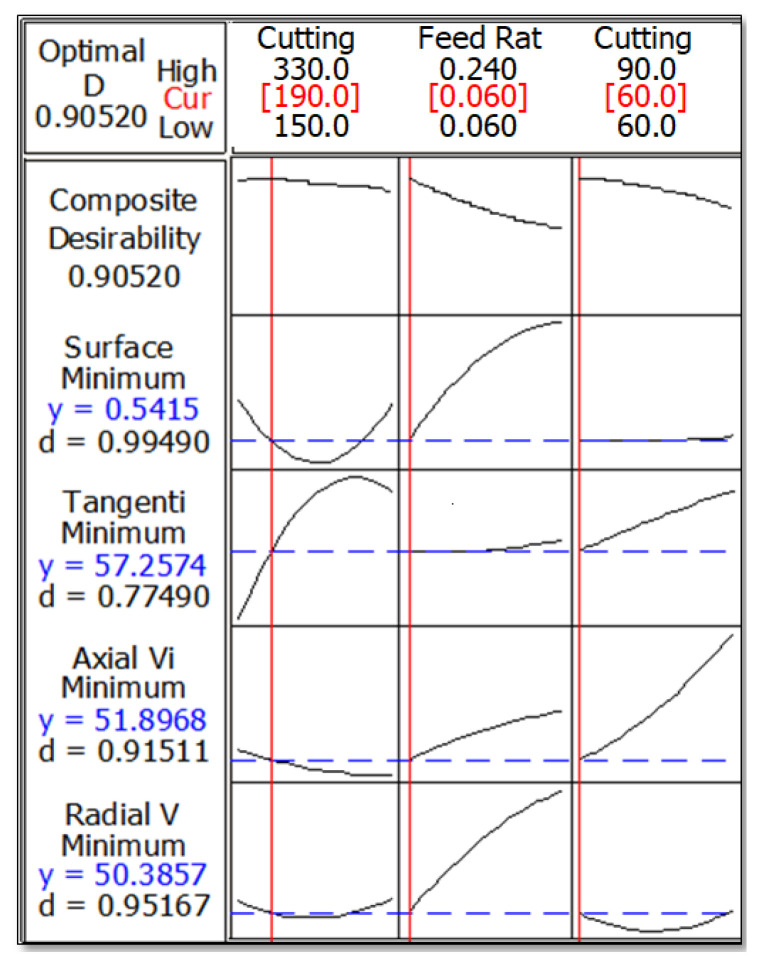
Optimum cutting conditions for minimum surface roughness and vibration.

Figure 7 indicates the optimum cutting conditions and related optimized response parameters. High and low show the boundary conditions for cutting conditions and the optimum values are marked in red. *y* indicates achieved optimum value while *d* implies the desirability for each parameter. According to results, *V* = 190 m/min, *f* = 0.06 mm/rev and *κ* = 60° should be selected for optimization. As it was stated before, a multi-criteria optimization approach was chosen to obtain minimum surface roughness and vibration components. It was reported that low feed rate and high cutting speed should be selected for minimum surface roughness [35]. Despite there being various studies regarding finding desirable surface roughness based on optimum cutting parameters, it should be investigated further by introducing additional input parameters into the model. For example, cutting edge angle has been researched by very few studies in the past. Moreover, three components of vibration have not been simultaneously optimized before. In this study, RSM based optimization was implemented for multi-criteria optimization of surface roughness and vibration based on three input parameters (cutting speed, feed and cutting edge angle).

### 3.5. Confirmation Experiment

In Table 8 a comparison of the experimental and predicted results is shown for the surface roughness and vibration components. To check the validity of the model, it is necessary to compare the predicted values with experimental ones [17]. The obtained results are in good agreement with the experimental values which can be obtained from the acceptable error rates within 1–10%. This means that, within the tested range of input parameters, the model can be used to predict any combination of cutting speed, feed and cutting edge angle to calculate the surface roughness and vibration components with minimal error. Surface roughness measurement was carried out from the operator while vibration components were measured with a sensor which leads to the increased robustness of the vibration results.

## 4. Conclusions

The current study investigates the impact of three machining parameters namely cutting speed, feed rate and cutting edge angle on surface roughness and vibration components during the turning of AISI 5140 steel. The vibration components were measured during the machining process while surface roughness data were collected after the end of each turning process. ANOVA based statistical analysis, graphical representation of affecting parameters, quadratic regression models and RSM based optimization were performed for surface roughness and vibration. There have been a number of studies in the open literature on the machinability of AISI 5140 steels to evaluate the machining parameters and their effect on the quality of the machined part. However, the three components of machining vibration were statistically analyzed for the first time in the open literature. In addition, the effect of the cutting edge angle which is rarely reported in the open literature was investigated to evaluate its impact on surface roughness and vibration components. Multi-criteria optimization, simultaneous optimization of surface roughness and vibration components were carried out to systematically predict the adequacy of the developed regression models and additional turning tests were carried out to validate the accuracy of the models. According to these examinations, the following conclusions can be made:

- Feed rate was found to be the parameter effective on surface roughness (69.4%) and axial vibration (65.8%), meanwhile cutting edge angle (75.5%) and cutting speed (64.7%) were dominant factors on radial vibration and tangential vibration, respectively.
- Among the three vibration components axial vibration was observed as the primary source of information for surface roughness. According to RSM, surface roughness and axial vibration can be optimized with remarkably high desirability of about 99% and 95%, respectively.
- The optimum results were found to be *V_c_* = 190 m/min, *f* = 0.06 mm/rev and *κ* = 60° to obtain minimum surface roughness and three components of vibration.
- RSM based quadratic regression models were obtained with 95%, 98%, 97% and 92% accuracy of surface roughness, tangential vibration, radial vibration and axial vibration. These results indicated the accuracy and reliability of the model which can be utilized for turning AISI 5140 steel.
- The predicted results regarding surface roughness and vibration were verified with an additional confirmation experiment. The comparison showed that there is a good agreement between the predicted and measured results with less than 10% error.
- The proposed methodology contains modeling and optimization for better machinability in the complex nature of turning.
- As a result, statistically reliable and optimum cutting conditions and vibration leading to best surface roughness were presented.

## Figures and Tables

**Table 1:** The most influential parameters in different studies on turning steel alloys.

	**Surface Roughness**							
**Reference**	**Material**	**Feed Rate**	**Cutting Speed**	**Nose Radius**	**Depth of Cut**	**Cooling Condition**	**Cutting Edge Angle**	**Optimization/Statistical Study**
[12]	AISI 5140	1th	3th	-	-	-	2th	ANOVA
[14]	AISI 410	1th	3th	2th	4th	-	-	Response Surface Methodology
[35]	Composites	1th	2th	-	-	-	3th	ANOVA
[36]	AISI 1040	3th	1th	-	2th	-	-	Response Surface Methodology
[41]	AISI 52100	1th	2th	-	3th	-	-	Response Surface Methodology
[42]	Hadfield	4th	2th	1th	3th	-	-	ANOVA, Response Surface Methodology
[34]	AISI 1050	1th	3th	-	4th	2th	-	ANOVA, Response Surface Methodology
	**Vibration Components**							
**Reference**	**Material**	**Feed Rate**	**Cutting Speed**	**Nose Radius**	**Depth of Cut**	**Hardness**	**Cutting Edge Angle**	**Optimization/Statistical Study**
[12]	AISI 5140	1th	3th	-	-	-	2th	ANOVA
[13]	AISI 5140	4th	2th	-	3th	-	1th	ANOVA
[28]	AISI 4140	2th	3th	-	4th	1th	-	ANOVA
[45]	AISI D2	-	1th	-	-	-	-	-

**Table 2:** The chemical composition of AISI 5140 carbon steel [12].

Element	C	Mn	Si	Cr	Ni	Mo	V	S	Cu	P
%	0.45	0.7	0.28	0.85	0.14	0.05	0.029	0.065	0.01	0.02

**Table 3:** Cutting parameters and factor levels [12].

Symbol	Parameters	Level 1	Level 2	Level 3
*V_c_*	Cutting Speed (m/min)	150	200	330
*f*	Feed Rate (mm/rev)	0.06	0.12	0.24
*κ*	Cutting edge angle (°)	60	75	90

**Table 4:** The experimental design and results.

Experiment Number	Design Parameters			Quality Indicators			
	Feed Rate *f* (mm/rev)	Cutting Speed *V_c_* (m/min)	Cutting Edge Angle *κ* (°)	Surface Roughness *R_a_* (µm)	Tangential Vibration *V_t_* (Hz)	Radial Vibration *V_r_* (Hz)	Axial Vibration *V_a_* (Hz)
1	0.06	150	60	0.78	54.88	51.25	50.09
2	0.12	150	60	1.7	54.6	54.62	52.72
3	0.24	150	60	2.15	54.63	57.86	54.89
4	0.06	200	60	0.69	57.96	51.12	50.88
5	0.12	200	60	0.95	56.85	53.25	52
6	0.24	200	60	1.8	58.98	55.45	53.12
7	0.06	330	60	0.81	60.44	50.03	50.5
8	0.12	330	60	1.74	59.1	52.74	52.9
9	0.24	330	60	1.96	60.5	53.85	55.1
10	0.06	150	75	0.108	53.12	57.89	50.06
11	0.12	150	75	0.17	54.45	58.47	51.09
12	0.24	150	75	0.244	55.42	60.74	54.71
13	0.06	200	75	0.429	59.88	56.87	50.6
14	0.12	200	75	0.745	60.1	57.47	51.2
15	0.24	200	75	0.202	61.5	59.52	53.66
16	0.06	330	75	0.432	66.98	55.96	51.1
17	0.12	330	75	0.214	65.88	56.14	52.78
18	0.24	330	75	0.6	66.9	56.98	53.89
19	0.06	150	90	0.108	55.41	61.86	50.6
20	0.12	150	90	0.17	56.1	64.89	51.2
21	0.24	150	90	0.244	56.7	69.11	53.66
22	0.06	200	90	0.429	61.12	60.42	50
23	0.12	200	90	0.745	63.55	63.87	52.9
24	0.24	200	90	0.202	64.12	68.99	53.9
25	0.06	330	90	0.432	69.5	59.85	53.1
26	0.12	330	90	0.214	70.2	62.41	55.01
27	0.24	330	90	0.6	71.5	67.88	56.8

**Table 5:** ANOVA results for surface roughness.

Cutting Parameters	Degree of Freedom	Sum of Squares	Mean Square	*F* Value	*p*-Value	Percent Contribution (%)
Surface Roughness *R_a_* (µm)						
Cutting Speed	1	0.5053	0.15356	5.28	0.034	5
Feed Rate	1	7.1810	7.28679	250.73	0.000	69.4
Cutting Edge Angle	1	0.2178	0.30487	10.49	0.005	2.1
Cutting Speed × Cutting Speed	1	1.3636	1.36355	46.92	0.000	13.2
Feed Rate × Feed Rate	1	0.3520	0.35203	12.11	0.003	3
Cut. Ed. Ang. × Cut. Ed. Ang.	1	0.0015	0.00145	0.05	0.826	0.01
Cutting Speed × Feed Rate	1	0.0006	0.00061	0.02	0.886	0.01
Cutting Speed × Cut. Ed. Ang.	1	0.2089	0.20886	7.19	0.016	2
Feed Rate × Cut. Ed. Ang.	1	0.0190	0.1903	0.65	0.430	0.1
Error	17	0.4941	0.02906			5
Total	26	10.3437				100

**Table 6:** ANOVA results for 3 components of vibration.

Cutting Parameters	Degree of Freedom	Sum of Squares	Mean Square	*F* Value	*p*-Value	Percent Contribution (%)
*Tangential Vibration V_t_* (Hz)						
Cutting Speed	1	474.602	497.769	608.01	0.000	64.7
Feed Rate	1	7.504	6.102	7.45	0.014	1
Cutting Edge Angle	1	140.337	170.937	208.79	0.000	19.1
Cutting Speed × Cutting Speed	1	34.157	34.157	41.72	0.000	4.6
Feed Rate × Feed Rate	1	0.319	0.319	0.39	0.541	0.001
Cut. Ed. Ang. × Cut. Ed. Ang.	1	0.100	0.100	0.12	0.731	0.001
Cutting Speed × Feed Rate	1	0.197	0.197	0.24	0.630	0.001
Cutting Speed × Cut. Ed. Ang.	1	60.596	60.596	74.02	0.000	8.2
Feed Rate × Cut. Ed. Ang.	1	1.709	1.709	2.09	0.167	0.2
Error	17	13.918	13.918	0.819		1.8
Total	26	733.438				100
*Radial Vibration V_r_* (Hz)						
Cutting Speed	1	23.109	25.211	19.27	0.000	3.2
Feed Rate	1	113.401	105.825	80.88	0.000	15.7
Cutting Edge Angle	1	545.711	539.891	412.62	0.000	75.5
Cutting Speed × Cutting Speed	1	1.078	1.078	0.82	0.377	0.1
Feed Rate × Feed Rate	1	0.909	0.909	0.69	0.416	0.1
Cut. Ed. Ang. × Cut. Ed. Ang.	1	6.948	6.948	5.31	0.034	1
Cutting Speed × Feed Rate	1	1.317	1.317	1.01	0.330	0.2
Cutting Speed × Cut. Ed. Ang.	1	0.113	0.113	0.09	0.772	0.001
Feed Rate × Cut. Ed. Ang.	1	7.901	7.901	6.04	0.025	1.1
Error	17	22.244	22.244	1.038		3
Total	26	722.729				100
*Axial Vibration V_a_* (Hz)						
Cutting Speed	1	10.4905	7.8527	17.88	0.001	11.7
Feed Rate	1	58.8353	57.2322	130.35	0.000	65.8
Cutting Edge Angle	1	1.3723	2.3810	5.42	0.032	1.5
Cutting Speed × Cutting Speed	1	1.1899	1.1899	2.71	0.118	1.3
Feed Rate × Feed Rate	1	1.1070	1.1070	2.52	0.131	1.2
Cut. Ed. Ang. × Cut. Ed. Ang.	1	2.3188	2.3188	5.28	0.035	2.6
Cutting Speed × Feed Rate	1	0.0979	0.0979	0.22	0.643	0.1
Cutting Speed × Cut. Ed. Ang.	1	6.3763	6.3763	14.52	0.001	7.1
Feed Rate × Cut. Ed. Ang.	1	0.0687	0.0687	0.16	0.697	0.001
Error	17	7.4642	7.4642	0.4391		8.3
Total	26	89.3209				100

**Table 7:** Response surface methodology parameter design and predicted responses.

Parameter	Goal	Lower	Target	Upper	Weight	Import	Predicted Value	Desirability
Surface Roughness	Min.	0.53	0.53	2.78	1	1	0.5415	0.99
Tangential Vibration	Min.	53.12	53.12	71.5	1	1	57.25	0.77
Radial Vibration	Min.	50.3	50.3	69.11	1	1	51.89	0.91
Axial Vibration	Min.	50.06	50.06	56.80	1	1	50.38	0.95
Desirability	-	-	-	-	-	-	-	0.90

**Table 8:** Comparison of experimental and predicted results.

Experimental Result	Predicted Value	Experimental Value	Accuracy	Error
Surface Roughness	0.5415 µm	0.6 µm	90%	10%
Tangential Vibration	57.25 Hz	58.15 Hz	99%	1%
Radial Vibration	51.89 Hz	50.99 Hz	99%	1%
Axial Vibration	50.38 Hz	51.39 Hz	99%	1%

## Nomenclature

ANOVA	Analysis of Variance
ANN	Artificial Neural Network
AISI	American Iron and Steel Institute
RSM	Response Surface Methodology
*R_a_*	Arithmetic Mean Value of Profile (µm)
*V_t_*	Tangential Vibration (Hz)
*V_a_*	Axial Vibration (Hz)
*V_r_*	Radial Vibration (Hz)
*p*	Probability of Significance
*F*	Variance Ratio
MS	Mean of Squares
SS	Sum of Squares
DF	Degree of Freedom
*V_c_*	Cutting Speed (m/min)
*κ*	Cutting edge angle (°)
*f*	Feed Rate (mm/rev)

## References

[1] Alsaran A. (2002). Determination of tribological properties of ion-nitrided AISI 5140 steel. Mater. Charact..

[2] Alsaran A., Çelik A. (2001). Structural characterization of ion-nitrided AISI 5140 low-alloy steel. Mater. Charact..

[3] Alsaran A., Karakan M., Celik A. (2002). The investigation of mechanical properties of ion-nitrided AISI 5140 low-alloy steel. Mater. Charact..

[4] Grzesik W. (2009). Wear development on wiper Al2O3–TiC mixed ceramic tools in hard machining of high strength steel. Wear.

[5] Ebrahimi A., Moshksar M. (2009). Evaluation of machinability in turning of microalloyed and quenched-tempered steels: Tool wear, statistical analysis, chip morphology. J. Mater. Process. Technol..

[6] Ebrahimi A., Moshksar M. (2007). Study of machinability in boring operation of microalloyed and heat-treated alloy steels. Mater. Sci. Eng. A.

[7] Li H.-Y., He H.-B., Han W.-Q., Yang J., Gu T., Li Y.-M., Lyu S.-K. (2015). A study on cutting and tribology performances of TiN and TiAlN coated tools. Int. J. Precis. Eng. Manuf..

[8] Zhang Y., Cheng Y., Hu H., Yin Z. (2017). Experimental study on cutting performance of microwave sintered Ti (C, N)/Al_2_O_3_ cermet tool in the dry machining of hardened steel. Int. J. Adv. Manuf. Technol..

[9] Huang X., Ren Y., Li T., Zhou Z., Zhang G. (2018). Influence of minimum quantity lubrication parameters on grind-hardening process. Mater. Manuf. Process..

[10] Grzesik W., Wanat T. (2005). Comparative assessment of surface roughness produced by hard machining with mixed ceramic tools including 2D and 3D analysis. J. Mater. Process. Technol..

[11] Kahraman F. (2017). Optimization of cutting parameters for surface roughness in turning of studs manufactured from AISI 5140 steel using the Taguchi method. Mater. Test..

[12] Kuntoğlu M., Aslan A., Sağlam H., Pimenov D.Y., Giasin K., Mikolajczyk T. (2020). Optimization and Analysis of Surface Roughness, Flank Wear and 5 Different Sensorial Data via Tool Condition Monitoring System in Turning of AISI 5140. Sensors.

[13] Aslan A. (2020). Optimization and Analysis of Process Parameters for Flank Wear, Cutting Forces and Vibration in Turning of AISI 5140: A Comprehensive Study. Measurement.

[14] Makadia A.J., Nanavati J. (2013). Optimisation of machining parameters for turning operations based on response surface methodology. Measurement.

[15] Mia M., Dhar N.R. (2016). Prediction of surface roughness in hard turning under high pressure coolant using Artificial Neural Network. Measurement.

[16] Nieslony P., Krolczyk G., Wojciechowski S., Chudy R., Zak K., Maruda R. (2018). Surface quality and topographic inspection of variable compliance part after precise turning. Appl. Surf. Sci..

[17] Kuntoğlu M., Sağlam H. (2019). Investigation of progressive tool wear for determining of optimized machining parameters in turning. Measurement.

[18] Ranjan J., Patra K., Szalay T., Mia M., Gupta M.K., Song Q., Krolczyk G., Chudy R., Pashnyov V.A., Pimenov D.Y. (2020). Artificial Intelligence-Based Hole Quality Prediction in Micro-Drilling Using Multiple Sensors. Sensors.

[19] Aslan A., Güneş A., Salur E., Şahin Ö.S., Karadağ H.B., Akdemir A. (2018). Mechanical properties and microstructure of composites produced by recycling metal chips. Int. J. Miner. Metall. Mater..

[20] Uzun M., Usca U.A. (2018). Effect of Cr particulate reinforcements in different ratios on wear performance and mechanical properties of Cu matrix composites. J. Braz. Soc. Mech. Sci. Eng..

[21] Şahin Ö.S., Güneş A., Aslan A., Salur E., Karadağ H.B., Akdemir A. (2019). Low-velocity impact behavior of porous metal matrix composites produced by recycling of bronze and iron chips. Iran. J. Sci. Technol. Trans. Mech. Eng..

[22] Aslan A., Salur E., Gunes A., Sahin O., Karadag H., Akdemir A. (2019). The mechanical properties of composite materials recycled from waste metallic chips under different pressures. Int. J. Environ. Sci. Technol..

[23] Pimenov D.Y., Abbas A.T., Gupta M.K., Erdakov I.N., Soliman M.S., El Rayes M.M. (2020). Investigations of surface quality and energy consumption associated with costs and material removal rate during face milling of AISI 1045 steel. Int. J. Adv. Manuf. Technol..

[24] Da Silva R.B., Sales W.F., Costa E.S., Ezugwu E.O., Bonney J., Da Silva M.B., Machado Á.R. (2017). Surface integrity and tool life when turning of Ti-6Al-4V with coolant applied by different methods. Int. J. Adv. Manuf. Technol..

[25] Neşeli S., Yaldız S., Türkeş E. (2011). Optimization of tool geometry parameters for turning operations based on the response surface methodology. Measurement.

[26] Kuntoğlu M., Sağlam H. An Alternative Method for Forming of Rifling Marks on Firearms: Ball Rolling. Proceedings of the IDEFIS.

[27] Abbas A.T., Ragab A.E., Al Bahkali E.A., El Danaf E.A. (2016). Optimizing cutting conditions for minimum surface roughness in face milling of high strength steel using carbide inserts. Adv. Mater. Sci. Eng..

[28] Prasad B.S., Babu M.P. (2017). Correlation between vibration amplitude and tool wear in turning: Numerical and experimental analysis. Eng. Sci. Technol. Int. J..

[29] Wojciechowski S., Twardowski P., Pelic M., Maruda R., Barrans S., Krolczyk G. (2016). Precision surface characterization for finish cylindrical milling with dynamic tool displacements model. Precis. Eng..

[30] Wang H., To S., Chan C. (2013). Investigation on the influence of tool-tip vibration on surface roughness and its representative measurement in ultra-precision diamond turning. Int. J. Mach. Tools Manuf..

[31] He C., Zong W., Zhang J. (2018). Influencing factors and theoretical modeling methods of surface roughness in turning process: State-of-the-art. Int. J. Mach. Tools Manuf..

[32] Salur E., Aslan A., Kuntoglu M., Gunes A., Sahin O.S. (2019). Experimental study and analysis of machinability characteristics of metal matrix composites during drilling. Compos. Part B Eng..

[33] Salur E., Aslan A., Kuntoğlu M., Güneş A., Şahin Ö.S. (2020). Optimization of Cutting Forces During Turning of Composite Materials. Acad. Platf. J. Eng. Sci..

[34] Sarıkaya M., Güllü A. (2014). Taguchi design and response surface methodology based analysis of machining parameters in CNC turning under MQL. J. Clean. Prod..

[35] Kumar R., Chauhan S. (2015). Study on surface roughness measurement for turning of Al 7075/10/SiCp and Al 7075 hybrid composites by using response surface methodology (RSM) and artificial neural networking (ANN). Measurement.

[36] Yadav R.N. (2017). A hybrid approach of Taguchi-Response surface methodology for modeling and optimization of duplex turning process. Measurement.

[37] Joardar H., Das N., Sutradhar G., Singh S. (2014). Application of response surface methodology for determining cutting force model in turning of LM6/SiCP metal matrix composite. Measurement.

[38] Noordin M., Venkatesh V., Sharif S., Elting S., Abdullah A. (2004). Application of response surface methodology in describing the performance of coated carbide tools when turning AISI 1045 steel. J. Mater. Process. Technol..

[39] Thomas M., Beauchamp Y., Youssef A., Masounave J. (1996). Effect of tool vibrations on surface roughness during lathe dry turning process. Comput. Ind. Eng..

[40] Sajjady S., Abadi H.N.H., Amini S., Nosouhi R. (2016). Analytical and experimental study of topography of surface texture in ultrasonic vibration assisted turning. Mater. Des..

[41] Bouacha K., Yallese M.A., Mabrouki T., Rigal J.-F. (2010). Statistical analysis of surface roughness and cutting forces using response surface methodology in hard turning of AISI 52100 bearing steel with CBN tool. Int. J. Refract. Met. Hard Mater..

[42] Horng J.-T., Liu N.-M., Chiang K.-T. (2008). Investigating the machinability evaluation of Hadfield steel in the hard turning with Al2O3/TiC mixed ceramic tool based on the response surface methodology. J. Mater. Process. Technol..

[43] Parida A.K., Maity K. (2019). Modeling of machining parameters affecting flank wear and surface roughness in hot turning of Monel-400 using response surface methodology (RSM). Measurement.

[44] Abbas A.T., Ragab A.E., Benyahia F., Soliman M.S. (2018). Taguchi Robust Design for Optimizing Surface Roughness of Turned AISI 1045 Steel Considering the Tool Nose Radius and Coolant as Noise Factors. Adv. Mater. Sci. Eng..

[45] Özbek O., Saruhan H. (2020). The effect of vibration and cutting zone temperature on surface roughness and tool wear in eco-friendly MQL turning of AISI D2. J. Mater. Res. Technol..

[46] Plaza E.G., López P.N. (2018). Application of the wavelet packet transform to vibration signals for surface roughness monitoring in CNC turning operations. Mech. Syst. Signal Process..

[47] Abouelatta O., Madl J. (2001). Surface roughness prediction based on cutting parameters and tool vibrations in turning operations. J. Mater. Process. Technol..

[48] Risbood K., Dixit U., Sahasrabudhe A. (2003). Prediction of surface roughness and dimensional deviation by measuring cutting forces and vibrations in turning process. J. Mater. Process. Technol..

[49] Misaka T., Herwan J., Ryabov O., Kano S., Sawada H., Kasashima N., Furukawa Y. (2020). Prediction of surface roughness in CNC turning by model-assisted response surface method. Precis. Eng..

[50] Upadhyay V., Jain P., Mehta N. (2013). In-process prediction of surface roughness in turning of Ti–6Al–4V alloy using cutting parameters and vibration signals. Measurement.

[51] Hessainia Z., Belbah A., Yallese M.A., Mabrouki T., Rigal J.-F. (2013). On the prediction of surface roughness in the hard turning based on cutting parameters and tool vibrations. Measurement.

[52] Sahoo A.K., Sahoo B. (2012). Experimental investigations on machinability aspects in finish hard turning of AISI 4340 steel using uncoated and multilayer coated carbide inserts. Measurement.

[53] Balaji M., Rao K.V., Rao N.M., Murthy B. (2018). Optimization of drilling parameters for drilling of TI-6Al-4V based on surface roughness, flank wear and drill vibration. Measurement.

[54] Kwak J.-S. (2005). Application of Taguchi and response surface methodologies for geometric error in surface grinding process. Int. J. Mach. Tools Manuf..

[55] Kuntoğlu M. (2016). Prediction of Progressive Tool Wear and Cutting Tool Breakageusing Acoustic Emission and Cutting Force Signals in Turning.

[56] Knight W.A., Boothroyd G. (2005). Fundamentals of Metal Machining and Machine Tools.

[57] Mia M., Dey P.R., Hossain M.S., Arafat M.T., Asaduzzaman M., Ullah M.S., Zobaer S.T. (2018). Taguchi S/N based optimization of machining parameters for surface roughness, tool wear and material removal rate in hard turning under MQL cutting condition. Measurement.

[58] Krolczyk G., Legutko S., Nieslony P., Gajek M. (2014). Study of the surface integrity microhardness of austenitic stainless steel after turning. Teh. Vjesn..

